# The Trend of Voluntary Warnings in Electronic Nicotine Delivery System Magazine Advertisements

**DOI:** 10.3390/ijerph14010062

**Published:** 2017-01-10

**Authors:** Ce Shang, Frank J. Chaloupka

**Affiliations:** 1 Health Policy Center, Institute for Health Research and Policy, University of Illinois at Chicago, Chicago, IL 60608, USA; fjc@uic.edu; 2 Department of Economics, University of Illinois at Chicago, Chicago, IL 60608, USA

**Keywords:** ENDS warnings, marketing, magazine ads, FDA regulation

## Abstract

Some manufacturers of electronic nicotine delivery systems (ENDS) voluntarily carried health warnings in their advertisements. This study examined these voluntary warnings in magazine ads and plotted their trends between 2012 and early 2015. ENDS magazine ads were obtained through Kantar media and warnings were collected from the Chicago Public Library or the Trinkets and Trash surveillance system. The prevalence of voluntary warnings, warnings with the specific capitalized word “WARNING”, and MarkTen warnings were examined after being weighted using factors related to exposure between January 2012 and March 2015. Five brands (MarkTen, NJOY, MISTIC, and some Blu) carried warnings during the study period. The prevalence of warnings post 2012 that contained a description of nicotine did not significantly increase until the launch of MarkTen, which also happened several months before April 2014 when the U.S. food and drug administration (FDA) published its proposed deeming rule. In addition, none of these warnings met the criteria required by the FDA in the final rules. Voluntary warnings, particularly MarkTen warnings, significantly increased in ENDS magazine ads between 2014 and 2015. It is important to monitor how ENDS manufacturers will comply with the FDA regulation related to warnings and how this regulation will ultimately impact ENDS risk perceptions and use.

## 1. Introduction

The prevalence of the use of electronic nicotine delivery systems (ENDS) among U.S. high school students had grown from 1.5% in 2013 to 16% in 2015, surpassing the prevalence of conventional cigarette use in this population [1]. The Surgeon General’s report on e-cigarette use among youth and young adults calls for action to protect young people from using e-cigarettes as they pose long-term harm to brain development and respiratory health. Moreover, they are associated with increased probability of other tobacco use and addiction [2]. Among tobacco control policies, warnings are an effective means to inform about health risks [3]. However, the evidence on the effectiveness of ENDS warnings is very scarce to date [4,5,6,7,8,9,10,11]. In addition, despite voluntary warnings used by some ENDS brands, some of these warnings were considered to be weak and may in fact not be noticed by audiences [9,11].

In May 2016, the U.S. food and drug administration (FDA) issued the final deeming rules that extend its regulatory authority over tobacco products including ENDS [12]. This is an important step that the FDA took to regulate the manufacturing, distribution, and sales of all tobacco products at the federal level. In the final deeming rules, the FDA requires “the display of health warnings on cigarette tobacco, roll-your own tobacco, and covered tobacco product packages and in advertisements”. For covered products including ENDS, the required warning reads: “This product contains nicotine. Nicotine is an addictive chemical” [12]. The FDA further requires these warnings to “appear on at least 30 percent of the two principal display panels of the package and at least 20 percent of the area of the advertisement” [12]. For “print advertisements and other advertisements with a visual component”, the FDA further requires warnings to appear in at least 12-point font size and be conspicuous, surrounded by a rectangular border, and comply with other detailed requirements [12].

Manufacturers and producers have long anticipated and prepared for the deeming of tobacco products by the FDA. In April 2014, the FDA proposed a similar warning for ENDS and other covered products, which reads “This product contains nicotine derived from tobacco. Nicotine is an addictive chemical” (In the final rules, the FDA revised the content by dropping the following words “derived from tobacco”) [13]. Even before the deeming proposal, many ENDS companies had some voluntary warnings such as California’s Proposition 65 warning (see details in Appendix A, and https://www.trinketsandtrash.org/) [14]. Some ENDS manufacturers even went further to list more potential risks in their “self-regulated” voluntary warnings that far exceed the FDA-proposed warning [15,16]. For example, both MarkTen and MISTIC described in their warnings that nicotine use may have harmful health consequences (see Appendix A, https://www.trinketsandtrash.org/).

Once the FDA regulation is implemented, ENDS ads will be required to carry conspicuous warnings about nicotine in ENDS and its addictiveness. However, the effects of mandatory warnings on ENDS risk perception and use behaviors remain unknown [4,5,6,7,8,9,10,11]. In fact, there is a very limited body of research on the effectiveness of any ENDS warnings, including voluntary and hypothetical ones, in changing ENDS risk perceptions or behaviors. Several studies have been conducted in various experimental settings and found that warnings which state ingredients or health risks may increase perceptions of ENDS harmfulness and reduce e-cigarette craving and preference for ENDS [4,5,6,7,8,9,10,11]. However, Mays et al. (2016) also found that warnings in ads had little effect and that participants did not pay attention to warnings in print ads [9]. Moreover, when warnings describe the modified or reduced risks of ENDS as compared to those of cigarettes, the messages may be considered as advertisements rather than warnings by participants in focus group studies and may also reduce perceived harms of ENDS [5,10].

To the best of our knowledge, only two recent studies examined existing voluntary warnings [6,11]. Lee et al. (2016) found that participants who were exposed to MarkTen warnings were more likely to agree that ENDS contain dangerous chemicals and could be dangerous, compared with those who were not exposed to warnings [6]. Participants who used both cigarettes and ENDS also expressed that the MarkTen warning made them think about quitting ENDS use, suggesting that this warning may incentivize dual users to return to exclusive cigarette smoking [6]. Wackowski et al. (2016) conducted focus group interviews and found that participants considered MarkTen warnings to be stronger and more noticeable than VUSE’s [11]. Participants also had mixed views on FDA warnings: while they thought a warning about nicotine and its addictiveness is not necessarily strong, some of them, nonetheless, thought they could prevent youth and nonsmokers from using ENDS [11].

Regardless, exposure to voluntary ENDS warnings may have increased in recent years as ENDS advertising has been growing considerably [17,18,19,20,21,22], with expenditures increasing from nearly zero in 2010 to $39 million in 2013 [17]. Among these expenditures, print advertising accounted for the largest share [17]. Therefore, it is important to document how voluntary ENDS warnings in print have developed to inform the FDA about the marketing situation before the enforcement of FDA ENDS warnings. Moreover, one study by Mays et al. (2016) suggested that warnings in print ads did not draw the attention of participants, and thus had limited impact on perceptions or behaviors [9]. This finding suggests that more studies are needed to understand the manufacturers’ strategy behind putting out warnings and the consequences of these voluntary warnings [11,16]. Finally, data about voluntary warnings are needed to facilitate an evaluation of the effectiveness of FDA warnings and other potential warnings in the future.

This study collected ENDS magazine ads during January 2012 and March 2015, and examined the distribution of magazines that carried ENDS ads, as well as which brands had advertised in magazines. We further examined trends of voluntary warnings with different characteristics in these ads, and explored how manufacturers responded to anticipated regulation in their advertising practices in a market with big tobacco companies as emerging players [16].

## 2. Data and Methods

### 2.1. Data

#### 2.1.1. Magazine Ads from Kantar Media

Kantar Media compiled all paid advertising spaces and expenditure data from the Publishers Information Bureau, Inc. (PIB) (New York, NY, USA) [23,24], covering consumer magazines with more than 350 subscribers. Specifically, Kantar Media provided measures that included the size of the ad, units, and the percentage of the publication’s total circulation reached by the ad. Expenditures were reported based on the current gross open one-time rates, with premiums added for location and coloration. The occurrence of ENDS ads in magazines from October 2011 to March 2015 and their characteristics including unit (number of ads insertions), advertisement’s national equivalence (an index between 0 and 2 that measures the size of ads and percent of circulation reached by the ad), and expenditure were obtained. There were 907 ENDS advertisements in total during the study period.

#### 2.1.2. Contents of Magazine Ads

The content of these ads was collected by reading and scanning hard copies in the Chicago Public Library or through its magazine subscription at Zinio.com. The contents of 105 ads (12% out of 907) were obtained from the Trinkets and Trash surveillance system (https://www.trinketsandtrash.org/) that monitors and documents tobacco industry marketing. The scanned copies or screen shots of ads can be viewed on the Trinkets and Trash website. Although some tabloids, such as certain issues of *OK! Weekly* and *Star*, were not accessible from either the library or these websites, and thus 146 (16% out of 907) ads were not available when we collected the data, ads of the same brand in the same quarter but published in a different issue or a different magazine were always available. We further observed that, regardless of magazines, brands put out the same ads during the same period. Therefore, we assumed that these ads, or at least the warnings, would be identical to those that we could find in other issues or in other magazines or tabloids during the same period.

### 2.2. Methods

#### 2.2.1. Categorization of Voluntary Warnings

Because the FDA-required ENDS warnings to describe nicotine and its addictiveness, voluntary warnings were defined as messages in ads that clearly describe either the health risks of consuming nicotine or state that nicotine is addictive [12,13]. As a result, although the ads for Blu between mid-2012 and mid-2014 (see Appendix A) usually stated that they were not a smoking cession product and should not be sold to minors, they did not describe nicotine and its addictiveness, nor did they list potential health risks resulting from nicotine consumption. Moreover, without describing any potential risks of the product, they stated that the products were not intended to “treat, prevent, or cure any disease or condition”, which could be interpreted in a positive way if this message somehow is considered to show that companies are trustworthy [11]. As a result, we did not consider such messaging as warnings. Finally, using the criteria of containing description of either nicotine, its addictiveness, or its potential health risks, we identified brands that carried such warnings in their magazine ads and the publication dates of these ads.

#### 2.2.2. Measures

Measures were constructed using Stata Version 14.1 (StataCorp, College Station, TX, USA) in a dataset consisting of the 907 ENDS ads with information on their publication dates, magazine, brand, unit, national equivalence, and expenditure. A dichotomous variable was constructed to measure the prevalence of voluntary warnings among ads by coding brands with voluntary warnings as 0 and those without as 1. Therefore, the mean of this variable measures the prevalence of ads that contained warnings.

We further constructed two other dichotomous variables to measure the prevalence of warnings with various characteristics. Specifically, because the FDA warning starts with the capitalized word “WARNING” [12], we examined the prevalence of warnings that had this word in their messaging. We also constructed a dichotomous variable to measure the prevalence of MarkTen warnings, which were more noticeable (See Appendix A) [11]. The language used in the warnings and their font size and location in the ads were described and presented in the supplement Appendix A.

#### 2.2.3. Analyses

We first examined the characteristics of the ENDS ads sample (*N* = 709), including ENDS brands that had advertised in magazines, magazines that contained ENDS ads, and ad features including ad unit, national equivalence, and expenditure. We also examined the average prevalence of voluntary warnings in the study period, as well as the prevalence of warnings carrying the capitalized word “WARNING” and MarkTen warnings.

Next, in order to examine the overall trend of ENDS warnings in magazine ads, we aggregated the above constructs by month. Following previous literature [25,26], to better measure the effectiveness of and exposure to these warnings in magazine ads, we used ad units, national equivalence, and expenditure as weights when aggregating. This weighting mechanism captured the likelihood that readers were exposed to these ads [25,26]. Last, because publication frequency varies and exposure to ads in a longer time window may be more relevant to the behavioral or perception change, we constructed and examined the three-month moving averages in our final step, which were the means of the monthly aggregated warning measures of the current and preceding two months. For sensitivity analyses, we also plotted the following trends: trends without applying moving averages, un-weighted trends of three-month moving averages, and trends of six-month moving average.

## 3. Results

In Table 1, we show the magazines that had ENDS warnings in the study periods and brands that advertised in magazines. Consistent with previous reports [17,23], the data show that ENDS ads frequently appeared in magazines that were appealing to a diverse array of audiences, such as Rolling Stone (79 ads, 8.71%), Men’s journal (44 ads, 4.85%), Playboy (33 ads, 3.64%), Maxim (35 ads, 3.86%), various tabloids (notably Star, 112 ads, 12.35%), etc. They also had a moderate presence in women’s magazines like ELLE (10 ads, 1.10%) and Vogue (8 ads, 0.88%), and occasionally in LGBT magazines such as Out (6 ads, 0.66%). In the same period, ENDS ads were dominated by major brands such as Blu (406 or 44.76% were Blu Cigs; and 26 or 2.87% were Blu Plus Cigs), MarkTen (239 or 26.35% were MarkTen E-Vapor; and 108 or 11.91% were MarkTen), Fin (40, 4.41%), and MISTIC (40, 4.41%).

After closely examining all ENDS magazine ads, we identified the following five brands that we considered as carrying voluntary warnings: MarkTen, MISTIC, NJOY, and VUSE during the entire study period, and Blu before June 2012 and after October 2014. Despite different language for the warnings used in their ads, all five brands stated that the product contained nicotine, with additional information either about its addictive nature or about some health risks (see Appendix A). Early Blu ads used the California Proposition 65 warning, which described nicotine as causing birth defects or other reproductive harm (see Appendix A). The Blu warnings post-October 2014 used the same language that the FDA proposed and VUSE warnings used language that was very similar [9,11]. MISTIC and MarkTen went further and put out warnings that listed risks such as “causing dizziness” and “very toxic by inhalation” (see Appendix A). MISTIC and MarkTen throughout the study period, and Blu ads post-October 2014 also used the capitalized word “WARNING”. Among all these warnings, only the MarkTen warning used at least a 12-point font size and had a rectangular border surrounding the text. Nevertheless, none of these warnings occupied at least 20 percent of the area of the advertisement or appeared in the upper portion of the area, with the ones in NJOY ads being hardly noticeable based on our observation.

Table 1 shows that during the study period, 53.36% of magazine ads (MarkTen, MISTIC, NJOY, VUSE, and some Blu out of all ads) had warnings or specified nicotine in the products; 49.39% of ads (MISTIC, MarkTen, and post-October-2014 Blu out of all ads) had warnings that used the capitalized word “WARNING”; 38.29% were MarkTen ads. On average, the ads were one unit and had a national equivalence of 0.95, suggesting a reach of one-page ad after taking account of the reach of circulation. The average expenditure per ad was $165,938.

**Figure 1:**
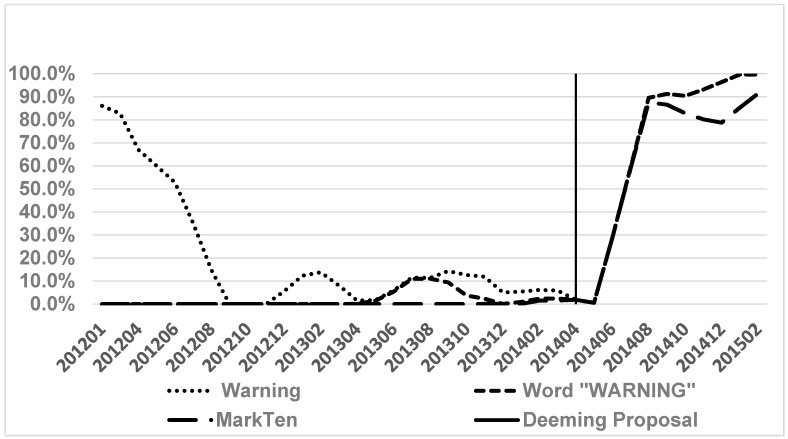
Warnings in electronic nicotine delivery systems (ENDS) Magazine Ads 2012–2015, three-month moving average (MA).

In Figure 1, we plot the trends of voluntary warnings in magazine ENDS ads between 2012 and early 2015, measured using the weighted prevalence of warnings, warnings with the specific capitalized word “WARNING”, and MarkTen warnings. The figure shows that during the study period, the prevalence of voluntary warnings in magazine ENDS ads had a somewhat inverted-U shape. That is, the warnings or ads that had nicotine information first decreased between January and September 2012, and then increased between June and December 2014. In addition, the prevalence of having the word “WARNING” had stayed close to 0 until early 2013, and significantly increased after MarkTen was introduced to the national market, which was in early 2014 [15,16]. In the first half of 2012, the prevalence of warnings or nicotine messaging dropped sharply. This was because Blu was purchased by Lorillard in April 2012 and since then had dropped the California proposition 65 warning from their magazine ads [15,16]. Between the acquisition of Blu by Lorillard and the launching of MarkTen (mid 2012–early 2014), the weighted prevalence of warnings or nicotine messaging in magazine ads was below 20% [16].

**Figure A1:**
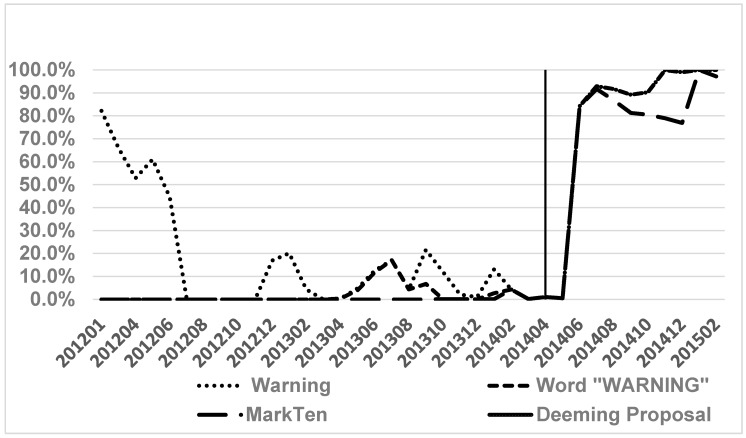
Warnings in ENDS Magazine Ads 2012–2015, weighted.

**Figure A2:**
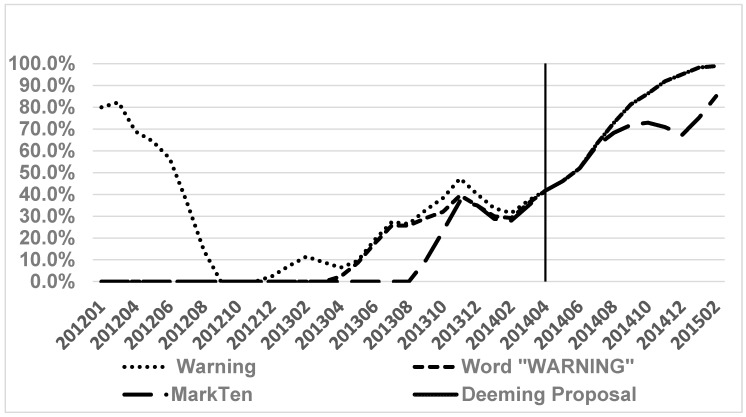
Warnings in ENDS Magazine Ads 2012–2015, three-month MA, un-weighted.

**Figure A3:**
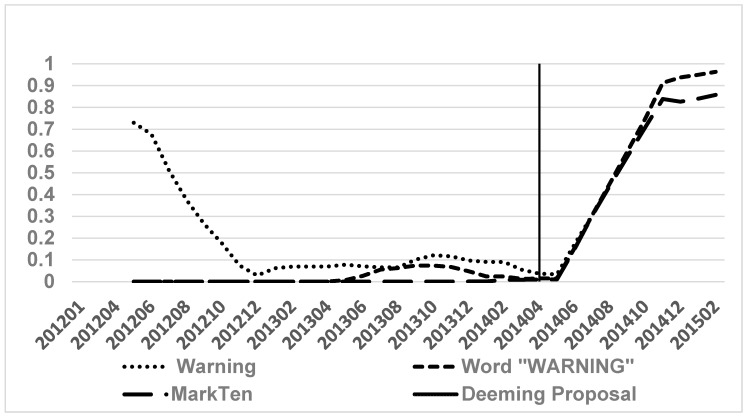
Warnings in ENDS Magazine Ads 2012–2015, six-month MA, weighted.

A marked increase in the prevalence of voluntary warnings in ENDS magazine ads had been observed since April 2014, when the FDA published its deeming proposal that outlined a plan to require warnings in ENDS ads [13]. Since then, MarkTen significantly increased their presence in magazines and Blu swiftly changed their warnings in ads to the FDA-proposed one [9,16]. As a result, the prevalence of voluntary warnings skyrocketed. While MarkTen ads dominated in magazines, brands other than MarkTen and Blu that also carried warnings did not actively advertise in magazines after the deeming proposal. Therefore, the trends of the several measures we constructed became almost identical since June 2014, which were de facto the trend of MarkTen and Blu warnings. As of March 2015, the overall prevalence of warnings in magazine ads had reached approximately 100%. We further examined the trend and its shape by conducting the sensitivity analyses we described previously in this study, and the results (Figure A1, Figure A2 and Figure A3) and conclusion remain very similar. The only difference worth noting is that if we did not apply weights, the trends took off around mid-2013, driven by MISTIC and NJOY, as well as by MarkTen, which started to advertise before its national launching in 2014 [16]. This difference further shows the importance of applying weights since the weighted measures, after taking account of exposure to ads, appeared to be more aligned with the marketing of ENDS companies.

## 4. Discussion

We examined the characteristics of ENDS voluntary warnings in magazine ads and plotted the trends of warning prevalence between January 2012 and March 2015. During this period, magazine ads for MarkTen, MISTIC, NJOY, VUSE, and some Blu ENDS contained language that at least stated that the product contains nicotine, with MarkTen, Blu (post-October 2014), and MISTIC warnings using the exact word “WARNING” and MarkTen and MISTIC warnings describing the potential health risks of nicotine use. In addition, Blu ads after October 2014 contained a warning that is identical to the one that the FDA proposed and VUSE ads contained a warning that is very similar.

Results show that, after using weighted measures reflecting ad exposure, the prevalence of voluntary ENDS warnings increased markedly after the FDA issued their deeming proposal in April 2014. Although Blu ads included the California proposition 65 warning in early 2012, this warning disappeared after the acquisition of Blu by Lorillard in mid-2012 [16]. Since then, the prevalence of voluntary warnings in magazine ads had been relatively low until the FDA deeming proposal. After the April 2014 deeming proposal, MarkTen dominated ENDS magazine advertising and has driven the prevalence of warnings to almost 100% as of March 2015.

This finding is not surprising given that ENDS magazine advertising was found to be dominated by Blu and MarkTen (Table 1), brands that were owned by big tobacco companies [16,17]. As many ENDS manufacturers had long anticipated the FDA to deem ENDS a tobacco product and regulate its production, distribution, and marketing [13], big tobacco companies may take this as an opportunity for marketing their ENDS products in a way that complies with future regulations. As a matter of fact, MarkTen, a brand owned by the Altria group, was launched not long before the FDA deeming proposal [27], with their magazine ads first seen in September 2013 and carrying the strongest warning labels to date (https://www.trinketsandtrash.org/) [11,17]. Blu, after being sold to Imperial Tobacco, swiftly switched its messaging in ads to the FDA-proposed warning by October 2014 (https://www.trinketsandtrash.org/). There has been speculation over why ENDS sold by big tobacco companies included strong warnings. One New York Times article published in September 2014 summarized various theories from “business strategy to appear more responsible” and “discouraging smokers from using a competing product”, to knowing that “many people don’t read the warnings anyway” [15,16]. To pinpoint the true intention falls out of the scope of this study. However, our research provides the following facts that may help future studies to better understand ENDS advertising at least in magazines. First, whether a brand included warnings in their magazine ads was not solely dependent on whether it was owned by a big tobacco company. For example, MISTIC, a brand not owned by any big tobacco company, used warnings that contain many potential risks of nicotine use (see Appendix A). In addition, Blu ads used to carry the California proposition 65 warning before its acquisition by Lorillard. Second, big tobacco companies did anticipate and respond swiftly to the FDA deeming. MarkTen has carried strong warnings since its introduction and Blu took only six months to complete the transition to carrying the FDA-proposed warning in all its ads. Thirdly, after the deeming proposal, many brands did not advertise in magazines as of March 2015, possibly awaiting the final rules by the FDA.

It is also worth noting that none of these warnings occupied 20% of the ad space or were placed on the top of the ads, and that only MarkTen used 12-point font size and had a rectangular border surrounding the warning text. In fact, based on our observation, almost all warnings except the MarkTen warning were in very small font and hardly noticeable. This finding poses questions as to whether these voluntary warnings were effective in altering risk perceptions and use behaviors. The study by Mays et al. (2016) suggests that although the warning statement itself may change perceived harm of ENDS and intention to try among nonsmokers, few participants paid attention to warnings in print ads [9]. As their warnings had a similar placement in ads and a greater size than those of the actual voluntary warnings in ads other than MarkTen ones, the warnings before MarkTen were likely not effective. Furthermore, warnings such as those in VUSE were considered by focus group participants to be “cool” and more like an ad, suggesting that the Blu and VUSE voluntary warnings may not stand out as warnings [11].

This focus group study and another study in an experimental setting both found that participants responded to MarkTen warnings by more likely perceiving harms of ENDS [6,11]. Our findings show that MarkTen, together with Blu, dominated ENDS magazine advertising. Therefore, it is important to analyze how MarkTen warnings affect the public’s risk perceptions of ENDS in future studies. Furthermore, our study provides important data predating the implementation of the FDA warning. The trends of warnings in this study illustrate that MarkTen started to advertise in mid-2013 and was aggressively advertising between 2014 and early 2015, which may have altered risk perceptions and ENDS use. Studies on the effects of the FDA final warning in such an environment are also needed.

There are several limitations of this study. First, as we previously described, we did not have access to all tabloid ads. However, given the dominance of big brands in magazine ads and that ads during the same period were unlikely to vary, this measurement error was very limited and unlikely influenced the overall trends. Second, the weighting mechanism did not capture the full array of factors that may impact consumers’ exposure to ads. Last, this study examined all forms of voluntary warnings in magazine ads, including those that were very small and hardly noticeable [9]. Therefore, the trends reported in this study do not reflect or measure the effectiveness of these warnings or exposure to these warnings.

## 5. Conclusions

In sum, this study illustrates the trends of voluntary warnings in ENDS magazine ads between January 2012 and March 2015. We found that MarkTen and Blu dominated ENDS magazine advertising and advertised in magazines that were appealing to a diverse array of audiences. Warnings that contained statements about nicotine were found in ads for MarkTen, MISTIC, NJOY, and VUSE, and in some Blu ads. The prevalence of these warnings, after weighted using factors related to exposure, significantly increased after the introduction of MarkTen. The prevalence of such warnings, warnings with the capitalized word “WARNING”, and MarkTen warnings greatly increased after April 2014, when the FDA published its deeming proposal. In addition, none of these warnings met the criteria required by the FDA in the final rules. It is important to monitor how ENDS manufacturers comply with the FDA regulations in the near future. Finally, in anticipation of a movement towards more conspicuous warnings with information of nicotine and its addictiveness, whether the required warning labels will impact the risk perceptions and ultimate use of ENDS and conventional cigarettes will need further assessment.

## Figures and Tables

**Table 1:** Characteristics of magazine ad sample, January 2012–March 2015 (*N* = 709).

**Section A, by Magazine Shares (43 Magazines)**					
**Magazines**	**Freq. (Percent)**	**Magazines**	**Freq. (Percent)**	**Magazines**	**Freq. (Percent)**
Allure	8 (0.88)	Hot Rod	5 (0.55)	Popular Mechanics	29 (3.2)
Automobile Magazine	8 (0.88)	In Style	12 (1.32)	Popular Science	17 (1.87)
Car and Driver	22 (2.43)	In Touch Weekly	33 (3.64)	Rolling Stone	79 (8.71)
Cosmopolitan	19 (2.09)	Life & Style Weekly	32 (3.53)	Soap Opera Digest	6 (0.66)
Country Weekly	11 (1.21)	Marie Claire	12 (1.32)	Southern Living	3 (0.33)
Cycle World	8 (0.88)	Maxim	35 (3.86)	Spin	4 (0.44)
Ebony	7 (0.77)	Men’s Journal	44 (4.85)	Sports Illustrated	40 (4.41)
Elle	10 (1.1)	Motor Trend	10 (1.1)	Star	112 (12.35)
Entertainment Weekly	61 (6.73)	National Enquirer	2 (0.22)	Time	12 (1.32)
ESPN Magazine	18 (1.98)	New York Magazine	3 (0.33)	TV Guide	37 (4.08)
Esquire	22 (2.43)	OK! Weekly	27 (2.98)	US Weekly	67 (7.39)
Essence	1 (0.11)	Out	6 (0.66)	Vanity Fair	9 (0.99)
Field & Stream	7 (0.77)	People	10 (1.1)	Vogue	8 (0.88)
Glamour	9 (0.99)	Playboy	33 (3.64)	Wired	1 (0.11)
GQ	8 (0.88)				
**Section B, by Brand Shares (18 Brands)**					
**Brands**	**Freq. (Percent)**	**Brands**	**Freq. (Percent)**	**Brands**	**Freq. (Percent)**
American Blue Tip	4 (0.44)	MarkTen E-Vapor	239 (26.35)	Swisher	1 (0.11)
Apollo	1 (0.11)	Markten	108 (11.91)	TRYST	5 (0.55)
Blu Cigs	406 (44.76)	Mistic	40 (4.41)	Vapir Rise	2 (0.22)
Blu Plus Cigs	26 (2.87)	Njoy King	15 (1.65)	Vapor Nation	1 (0.11)
Cigirex	2 (0.22)	Playboy Premium	1 (0.11)	VaporGenie	14 (1.54)
Fin	40 (4.41)	Starfire	1 (0.11)	Vuse	1 (0.11)
**Section C, Variables**					
**Variable**	**Percent**	**Variable**	**Mean**		
Any Warning **^1^**	53.36	Ad national equivalence	0.95		
“WARNING” **^1^**	49.39	Expenditure	165,938		
MarkTen **^1^**	38.29	Units	1.01		

**^1^** un-weighted prevalence was reported.

## Appendix A

Examples of language used in warnings by different ENDS brands:

MarkTen: *Rolling Stone* RS1214, 31 July 2014, a two-page spread ad in the magazine cover. Font size is approximately 12.

“WARNING: This is not intended for use by women who are pregnant or breastfeeding, or persons with or at risk of heart disease, high blood pressure, diabetes, or taking medicine for depression or asthma. Nicotine is addictive and habit forming, and it is very toxic by inhalation. Nicotine can increase your heart rate and blood pressure and cause dizziness, nausea, and stomach pain. Inhalation of this product may aggravate existing respiratory conditions.”

MISTIC: *Peoples’ Magazine*, 8 July 2013, a ½ page ad inside magazine with very small font.

“WARNING: MISTIC Electric Cigarettes are intended for use by smokers of legal smoking age (18 or older in California), and not by children, women who are pregnant or breastfeeding, or persons with or at risk of heart disease, high blood pressure, diabetes, or taking medication for depression or asthma. If you smoke tobacco products you are encouraged to stop. Identification of all persons under 26 will be required before purchase. MISTIC products are not a smoking cessation product and have not been tested as such. This product and the statements made within have not been evaluated by the U.S. Food and Drug Administration or any other international health or regulatory authority, unless otherwise noted in MISTIC’s materials. These statements and MISTIC product are not intended to diagnose, treat, cure or prevent any condition, disorder, disease or physical or mental conditions and should not be used as a substitute for your own physician’s advice. MISTIC products contain nicotine, a chemical known to the State of California to cause birth defects or other reproductive harm. Nicotine is addictive and habit forming, and is very toxic by inhalation, in contact with the skin, or if swallowed; danger of serious damages of health by prolonged exposure if swallowed, irritating to the eyes and skin; may cause sensitization by skin contact; may cause harm to the unborn child; vapors may cause drowsiness or dizziness; very toxic; very toxic to aquatic organisms, may cause long-term adverse effect in the aquatic environment. Ingestion of the non-vaporized concentrated ingredients in the cartridges can be poisonous. After contact with skin, wash immediately with plenty of water and seek medical advice; in case of accident or if you feel unwell, seek medical advice (show label when possible). This material and its container must be disposed of in a safe way; use appropriate containment to avoid environmental contamination.”

NJOY: *Out* magazine 1 October 2014, one-page ad with messaging in very small font and without stating warning specifically.

“@2013NJOY, Inc. Not for sale to minors. Contains Nicotine, which is addictive. Not a smoking cessation product. More information at http://www.njoy.com/pages/FAQs.html.”

VUSE: Playboy, January/February 2014, two-spread page with messaging in small font and without stating warning specifically.

“VUSE contains nicotine extracted from the tobacco plant. Nicotine is addictive and no tobacco product has been shown to be safe.”

Blu: Esquire 1 January 2012, one-page ad with a visible warning in small font.

“19+ only. CALIFORNIA PROPOSITION 65 Warning: this product contains nicotine, a chemical known to the state of California to cause birth defects or other reproductive harm.”

Examples of language used in messaging without risks or warnings:

Blu: People. 20 January 2014, one-page ad with messaging in very small font and without stating risks or warning specifically.

“NOT FOR SALE TO MINORS. blu eCigs^®^ electronic cigarettes are not a smoking cessation product and have not been evaluated by the Food and Drug Administration, nor are they intended to treat, prevent or cure any disease or condition. © 2013 LOEC, Inc., bluTM and blu eCigs^®^ are trademarks of Lorillard Technologies, Inc. (photograph by Francesco Carrozzini).”

**Supplement Figure: Sensitivity Analyses of Warning Strength in ENDS Magazine Ads, 2012–2015.**
